# Supplementary material for: Bivalirudin vs. Enoxaparin in Intubated COVID-19 Patients: A Pilot Multicenter Randomized Controlled Trial
Source: J Clin Med. 2022 Oct 11;11(20):5992. doi: 10.3390/jcm11205992 (PMC9604898; doi:10.3390/jcm11205992)
Supplement: Supplementary file 1 [file jcm-11-05992-s001.zip › jcm-1895887-supplementary.pdf]

Bivalirudin versus Enoxaparin in Intubated COVID-19 Patients: A Pilot Multicenter Randomized Controlled Trial

Eugenio Garofalo [1], Gianmaria Cammarota [2]; Giuseppe Neri [1]; Sebastiano Macheda [3], Eugenio Biamonte [1], Pino Pasqua [4]; Maria Laura Guzzo [5], Federico Longhini [1,*] and Andrea Bruni [1] on behalf of the BivaCOVID authors

1 Anesthesia and Intensive Care, Department of Medical and Surgical Sciences, "Magna Graecia" University, 88100 Catanzaro, Italy
2 Department of Anesthesia and Intensive Care Medicine, University of Perugia, 06121, Perugia, Italy
3 Anesthesia and Intensive Care Unit, Grande Ospedale Metropolitano, 89121 Reggio Calabria, Italy;
4 Anesthesia and Intensive Care Unit, Annunziata Hospital, 87100, Cosenza, Italy
5 Anesthesia and Intensive Care Unit, "Pugliese Ciaccio" Hospital, 88100, Catanzaro, Italy
* Correspondence: longhini.federico@gmail.com; Tel.: +39-34-7539-5967

Electronic Supplemental Material

Table S1. Clinical characteristics at day 3 from randomization

	Bivalirudin (n=29)	Enoxaparin (n=29)	P value
Hemodynamic			
Need for norepinephrine - n (%)	14 (48%)	15 (52%)	0.793
Norepinephrine (mcg*kg/min)	0.30 [0.20; 0.45]	0.30 [0.20; 0.50]	0.738
**Mechanical ventilation**			
Volume controlled - n (%)	14 (48%)	19 (66%)	0.289
Pressure support - n (%)	15 (52%)	10 (34%)	
Arterial Blood Gases			
pH	7.39 [7.37; 7.41]	7.38 [7.37; 7.41]	0.814
$PaCO_2$ (mmHg)	41.0 [38.3; 42.3]	41.2 [37.9; 42.4]	0.797
PaO_2/FiO_2	144 [124; 193]	150 [130; 186]	0.834
HCO_3 (mMol/L)	24.3 [23.0; 25.2]	24.6 [23.8; 25.5]	0.669
Lac (mMol/L)	1.7 [0.9; 2.7]	1.5 [0.8; 3.7]	0.785
Blood tests			
Platelets count	231 [170; 297]	245 [161; 307]	0.549
aPTT	60 [58; 63]	35 [32; 37]	<0.001
PT	12 [12; 13]	12 [12; 13]	0.121
PT-INR	1.15 [1.08; 1.21]	1.13 [1.09; 1.19]	0.737
D-dimer	8.13 [5.95; 12.29]	7.98 [5.67; 12.84]	0.911
Fibrinogen	412 [360; 552]	498 [416; 634]	0.063

Procalcitonin	6.54 [3.33; 19.57]	6.47 [1.95; 16.39]	0.544
C-Reactive Protein	98.4 [37.9; 176.6]	67.5 [27.8; 148.7]	0.635

Table S2. Clinical characteristics at day 7 from randomization

	**Bivalirudin (n=27)**	**Enoxaparin (n=28)**	**P value**
Hemodynamic			
Need for norepinephrine - n (%)	8 (30%)	11 (39%)	0.567
Norepinephrine (mcg*kg/min)	0.40 [0.20; 0.58]	0.50 [0.20; 0.60]	0.671
**Mechanical ventilation**			
Volume controlled - n (%)	4 (15%)	9 (32%)	0.177
Pressure Support - n (%)	18 (67%)	17 (61%)	
Non-Invasive Ventilation - n (%)	2 (7%)	2 (7%)	
High-Flow Nasal Cannula - n (%)	3 (11%)	0 (0%)	
Arterial Blood Gases			
pH	7.39 [7.38; 7.40]	7.39 [7.38; 7.40]	0.404
$PaCO_2$ (mmHg)	39.6 [38.8; 42.1]	40.1 [38.2; 42.3]	0.500
PaO_2/FiO_2	205 [160; 246]	174 [139; 194]	0.033
HCO_3 (mMol/L)	23.8 [23.4; 24.6]	24.2 [23.7; 25.1]	0.221
Lac (mMol/L)	0.9 [0.7; 1.8]	1.4 [0.9; 2.0]	0.192
Blood tests			
Platelets count	217 [187; 287]	246 [212; 286]	0.359
aPTT	63 [59; 66]	31 [30; 35]	<0.001
PT	12 [12; 13]	12 [12; 13]	0.512
PT-INR	1.15 [1.12; 1.21]	1.13 [1.06; 1.21]	0.171
D-dimer	6.43 [4.12; 8.65]	7.84 [4.35; 11.90]	0.222
Fibrinogen	427 [387; 522]	471 [391; 568]	0.325
Procalcitonin	4.23 [2.12; 10.54]	6.04 [2.93; 20.67]	0.368
C-Reactive Protein	31.2 [18.4; 68.9]	41.6 [21.2; 96.9]	0.723

Table S3. Clinical characteristics at day 15 from randomization

	**Bivalirudin (n=9)**	**Enoxaparin (n=18)**	**P value**
Hemodynamic			
Need for norepinephrine - n (%)	1 (11%)	7 (39%)	0.201
Norepinephrine (mcg*kg/min)	0.80 [0.80; 0.80]	0.70 [0.60; 0.80]	0.750
**Mechanical ventilation**			

Volume controlled - n (%)	1 (11%)	7 (39%)	0.209
Pressure Support - n (%)	0 (0%)	1 (6%)	
Non-Invasive Ventilation - n (%)	1 (11%)	0 (0%)	
High-Flow Nasal Cannula - n (%)	7 (78%)	10 (55%)	
Arterial Blood Gases			
pH	7.40 [7.38; 7.42]	7.38 [7.34; 7.42]	0.385
$PaCO_2$ (mmHg)	39.4 [35.3; 41.7]	37.3 [36.2; 39.0]	0.520
PaO_2/FiO_2	293 [229; 315]	226 [118; 280]	0.045
HCO_3 (mMol/L)	24.6 [23.4; 25.0]	23.9 [22.6; 24.5]	0.554
Lac (mMol/L)	0.70 [0.55; 1.10]	0.85 [0.70; 5.60]	0.147
Blood tests			
Platelets count	251 [184; 273]	263 [137; 325]	0.625
aPTT	59 [58; 62]	35 [33; 38]	<0.001
PT	13 [12; 14]	12 [12; 14]	0.303
PT-INR	1.16 [1.15; 1.28]	1.13 [1.10; 1.21]	0.226
D-dimer	3.78 [2.84; 7.99]	8.95 [3.85; 13.66]	0.111
Fibrinogen	401 [335; 453]	459 [375; 505]	0.227
Procalcitonin	5.12 [0.53; 6.67]	2.49 [1.13; 19.14]	0.938
C-Reactive Protein	23.8 [13.5; 42.3]	37.9 [21.4; 210.1]	0.208
